# Isolation and evaluation of erythroid progenitors in the livers of larval, froglet, and adult *Xenopus tropicalis*

**DOI:** 10.1242/bio.059862

**Published:** 2023-07-27

**Authors:** Kazuki Omata, Ikki Nomura, Akito Hirata, Yuka Yonezuka, Hiroshi Muto, Ryo Kuriki, Kirin Jimbo, Koujin Ogasa, Takashi Kato

**Affiliations:** ^1^ Department of Biology, School of Education, Waseda University, 2-2 Wakamatsu, Shinjuku, Tokyo 162-8480, Japan; ^2^ Graduate School of Advanced Science and Engineering, Waseda University, 2-2 Wakamatsu, Shinjuku, Tokyo 162-8480, Japan

**Keywords:** *Xenopus*, Erythropoietin receptor, Erythroid progenitor, Flow cytometry, Acridine orange

## Abstract

*Xenopus* liver maintains erythropoietic activity from the larval to the adult stage. During metamorphosis, thyroid hormone mediates apoptosis of larval-type erythroid progenitors and proliferation of adult-type erythroid progenitors, and a globin switch occurs during this time. In addition, the whole-body mass and the liver also change; however, whether there is a change in the absolute number of erythroid progenitors is unclear. To isolate and evaluate erythroid progenitors in the *Xenopus* liver, we developed monoclonal ER9 antibodies against the erythropoietin receptor (EPOR) of *Xenopus*. ER9 recognized erythrocytes, but not white blood cells or thrombocytes. The specificity of ER9 for EPOR manifested as its inhibitory effect on the proliferation of a *Xenopus* EPOR-expressing cell line. Furthermore, ER9 recognition was consistent with *epor* gene expression. ER9 staining with Acridine orange (AO) allowed erythrocyte fractionation through fluorescence-activated cell sorting. The ER9^+^ and AO-red (AOr)^high^ fractions were highly enriched in erythroid progenitors and primarily localized to the liver. The method developed using ER9 and AO was also applied to larvae and froglets with different progenitor populations from adult frogs. The liver to body weight and the number of ER9^+^ AOr^high^ cells per unit body weight were significantly higher in adults than in larvae and froglets, and the number of ER9^+^ AOr^high^ cells per unit liver weight was the highest in froglets. Collectively, our results show increased erythropoiesis in the froglet liver and demonstrate growth-dependent changes in erythropoiesis patterns in specific organs of *Xenopus*.

## Teaser

**Summary:** A fluorescence-activated cell sorting method was applied using specific antibodies against *Xenopus* erythroid progenitors. This method enables genotyping and evaluation of erythroid cells in the livers of *Xenopus sp*.

## INTRODUCTION

Erythropoiesis undergoes several changes to produce red blood cells (RBCs) depending on the developmental events, including switching hemoglobin (Hb) type. Fetal Hb has a higher affinity for oxygen than adult Hb, leading to an increased oxygen supply from the mother to the fetus (Stamatoyannopoulos, 2005). Globin switch is conserved among vertebrates, which enables them to cope with the environmental oxygen (Brownlie et al., 2003; Jurd and Maclean, 1969; Kobel and Wolff, 1983). The mechanism of the globin switch has been investigated in amphibians due to the advantage in the induction of metamorphosis by thyroid hormone. During metamorphosis in an African clawed frog, *Xenopus laevis*, thyroid hormone mediates apoptosis of larval-type erythroid progenitor cells and proliferation of adult-type erythroid progenitor cells (Tamori and Wakahara, 2000a), resulting in clonal replacement in erythroid cells. Consistently, thyroid hormone-mediated apoptosis of larval-type tissue occurs in the whole body, resulting in conversion to adult-type tissue, which results in nearly a 50% loss in body weight (BW) (Ishizuya-Oka et al., 2010; Tata, 1968). In the liver, hepatocyte alteration that includes gene expression to adult-type pattern occurs in a single population, which increases hepatocyte area clarifying the hepatic cord (Mukhi et al., 2010; Okui et al., 2016). The posterior of the three hepatic lobes undergoes a reduction in its mass. However, the other lobes increase in mass (Tamori and Wakahara, 2000a), indicating a change in the proportion of the liver to the whole body. The difference in the erythropoietic capacity in the metamorphic *Xenopus* liver can be implicated from these findings; however, the variation in the absolute number of erythroid progenitor cells has not yet been elucidated.

Erythropoietin (EPO) and its classical receptor (EPOR) regulate erythropoiesis by its specific cellular signaling (Krantz, 1991; D'Andrea and Zon, 1990). It has been conferred that the EPO-EPOR axis is widely conserved among vertebrates. In the *Xenopus* model, we have previously reported colony-forming erythroid progenitors under the stimulation of EPO (Nogawa-Kosaka et al., 2011) and EPO-responsive erythroid progenitors in the larval and adult liver (Okui et al., 2016). An *in-vitro* culture system with EPO can be used to observe cells with actual proliferation capacity; however, direct evidence for EPOR protein expression in these progenitors is lacking. Another issue is that the genotype of the cells stimulated *in vitro* does not fully reflect the genotype *in vivo*.

In addition, *in-situ* detection using erythroid-specific gene probes or antibodies can also analyze the localization of erythroid progenitors. However, it also detects mature erythrocytes because *Xenopus* erythrocytes maintain gene expression even after terminal maturation (Maclean et al., 1973). One solution to these problems is flow cytometry followed by fluorescence-activated cell sorting with a monoclonal antibody (MoAb) against a cell surface antigen, which allows quantitative analysis in addition to cell isolation. Therefore, we generated a novel MoAb against EPOR in the present study to isolate and evaluate *Xenopus* erythroid progenitors. This antibody also allowed us to examine whether erythroid lineage cells express EPOR protein.

Molecular biological analyses of aquatic anuran hematopoiesis have been performed using *X. laevis* because the species is easy to handle as an experimental animal and is tolerant to a wide range of living conditions. Along with *X. laevis*, which has allotetraploidy (Session et al., 2016), the use of its close diploid relative, *Xenopus tropicalis*, also has advantages in molecular-based analysis because it has fewer genes and more established lines than *X. laevis* (Hellsten et al., 2010; Igawa et al., 2015).

The present study investigated the expression of EPOR protein in *Xenopus* erythroid cells using an anti-EPOR antibody. In addition, we isolated and characterized erythroid progenitors in the liver of *X. tropicalis* and examined changes in their absolute numbers during metamorphosis.

## RESULTS

### Reactivity of ER3, ER9, and ER11 to peripheral blood cells of *X. laevis* and *X. tropicalis*

**Fig. 1.**
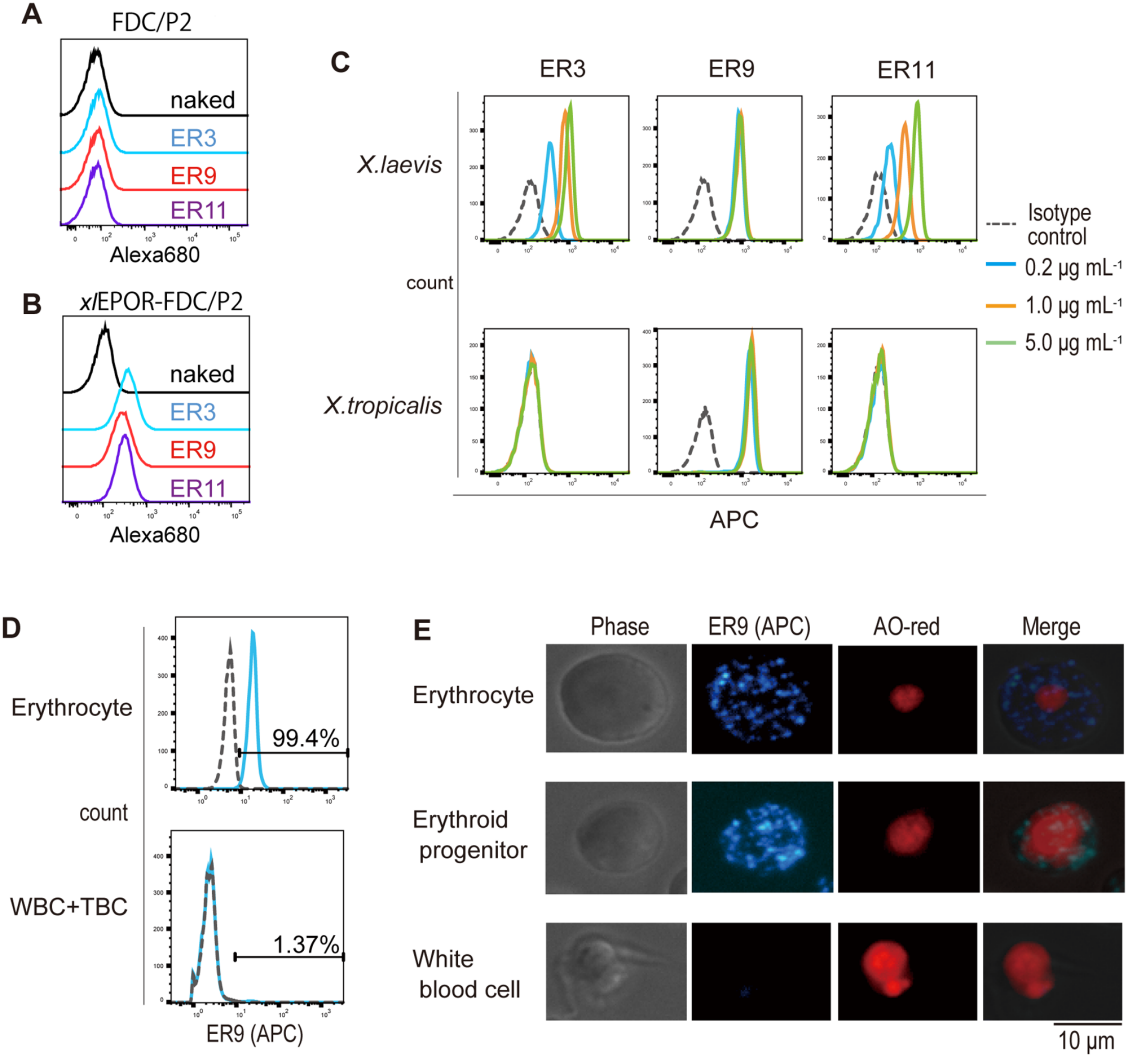
**Specificity of anti-*Xenopus laevis* erythropoietin receptor monoclonal antibody.** (A-B) Reactivities of ER3, ER9, and ER11 to FDC/P2 cell line (A) or *xl*EPOR-FDC/P2 cell line (B). Cells that did not react with the antibody were used as the negative control. A total of 50,000 cells were analyzed. (C) Reactivities of ER3, ER9, and ER11 to the peripheral blood cells of *X. laevis* or *X. tropicalis*. A mouse IgG2a isotype control was used as the negative control. Ten thousand cells were analyzed. (D) Reactivity of ER9 to peripheral erythrocytes (top) or thrombocytes and white blood cells (bottom). Ten thousand cells were analyzed. (E) Fluorescent microscopic images of the cells acquired after staining with ER9-APC (blue) and AO (red). Original images were obtained in ×40 magnification. APC, allophycocyanin; TBC, thrombocyte; WBC, white blood cell.

We established novel antibodies to *xl*EPOR that recognized *Xenopus* erythrocytes. ER3, ER9, and ER11 monoclonal antibodies did not recognize the FDC/P2 cell line (Fig. 1A). However, they showed reactivities to the FDC/P2 cell line forced to express *X. laevis* EPOR (*xl*EPOR-FDC/P2) (Fig. 1B), indicating that all three monoclonal antibodies recognize the *X. laevis* EPOR molecule. ER3, ER9, and ER11 recognized peripheral blood cells of *X. laevis*, though ER3 and ER11 were concentration-dependent (Fig. 1C). Only ER9 recognized blood cells from both *X. laevis* and *X. tropicalis*. Two species of *Xenopus, X. laevis* and *X. tropicalis,* are typically used as model organisms and were distinguished according to the study's aim. The present study focused on *X. tropicalis* because the sequence and expression pattern of the shorter homeologue of *X. laevis* EPOR, named *epor.S*, is currently unclear, and *epor.S* expression possibly affects the interpretation of recognition specificity or antigen expression. Moreover, ER9 did not recognize white blood cells and thrombocytes (Fig. 1D), suggesting its erythrocyte specificity; more than 99% of mature peripheral erythrocytes were ER9-recognizable.

### Neutralization of Xenopus EPO activity

**Fig. 2.**
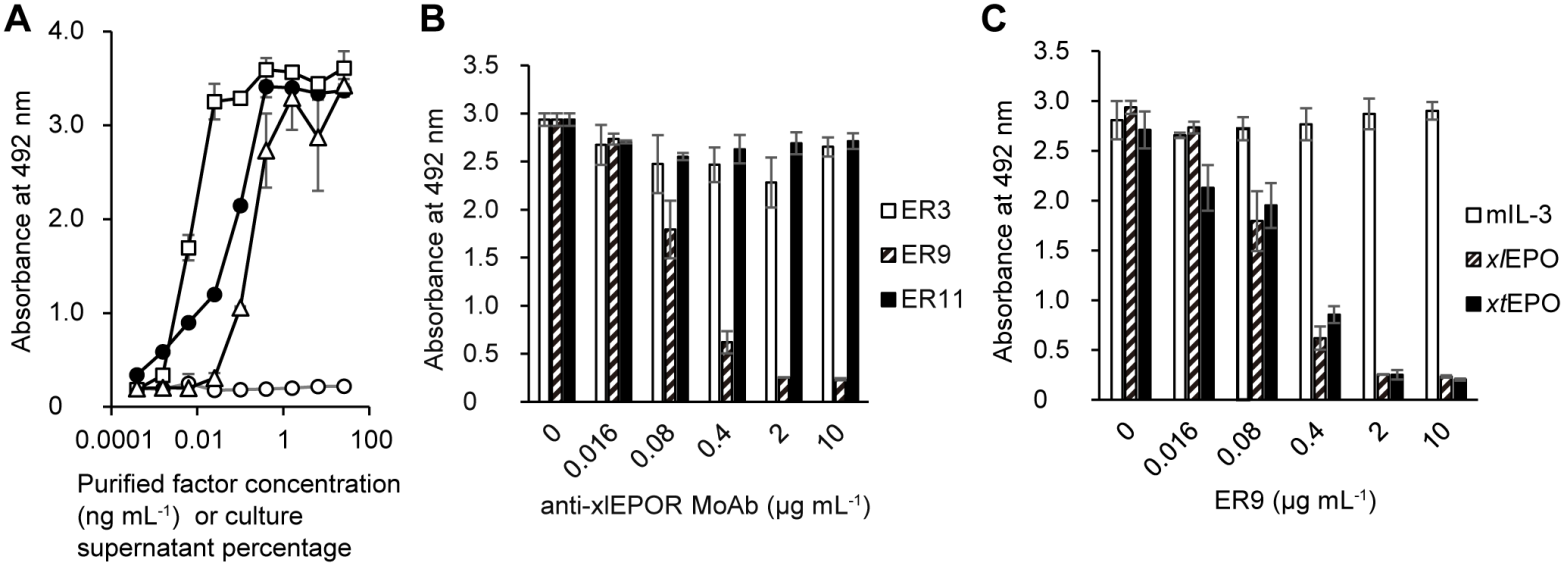
**Inhibition of *xl*EPOR-FDC/P2 proliferation by anti-*xl*EPOR monoclonal antibodies.** (A) Proliferative response of *xl*EPOR-expressing FDC/P2 cell line to rmIL-3, recombinant *xl*EPO, *xt*EPO, and the mock control in the 2-(4-iodophenyl)-3-(4-nitrophenyl)-5-(2,4-disulfophenyl)-2H-tetrazolium (WST-1) assay. Symbols: filled circle, mIL-3 (ng mL^−1^); open triangle, *xl*EPO (%); open square, *xt*EPO (%); open circle, mock control (%). (B) Inhibitory effects of anti-*xl*EPOR monoclonal antibodies on the proliferation of *xl*EPOR-FDC/P2 cells induced by *xl*EPO. The concentration of *xl*EPO was ten times the EC_50_ in *xl*EPOR-FDC/P2 proliferation. **(**C) Inhibitory effects of ER9 on the proliferation of *xl*EPOR-FDC/P2 cells induced by rmIL-3, *xl*EPO, and *xt*EPO. The concentration of each cytokine was ten times the EC_50_ in *xl*EPOR-FDC/P2 proliferation.

*xl*EPOR-FDC/P2 cell lines were used to test MoAbs for their ability to inhibit proliferation induced by *xl*EPO and *xt*EPO (Fig. 2A). ER9 completely inhibited proliferation, whereas ER3 and ER11 did not (Fig. 2B). ER9 also inhibited *xt*EPO-induced proliferation (Fig. 2C). None of the antibodies inhibited IL-3 activity, thus exhibiting specificity of inhibition.

The specificity of ER9 for EPOR was partially demonstrated as an inhibitory effect on EPO-induced proliferation and binding ability to *xl*EPOR-FDC/P2 cell line; however, we should be careful about the recognition specificity of EPOR antibodies because MoAbs to EPOR recognizing non-EPOR proteins have been found in humans (Elliott et al., 2006). Therefore, in the subsequent results, we do not refer to ER9-recognizable cells as EPOR-positive, but as ER9-positive (ER9^+^).

### Enrichment of erythroid progenitors from liver cells on the Percoll discontinuous density gradient and the reactivity of ER9 to erythroid progenitors

**Fig. 3.**
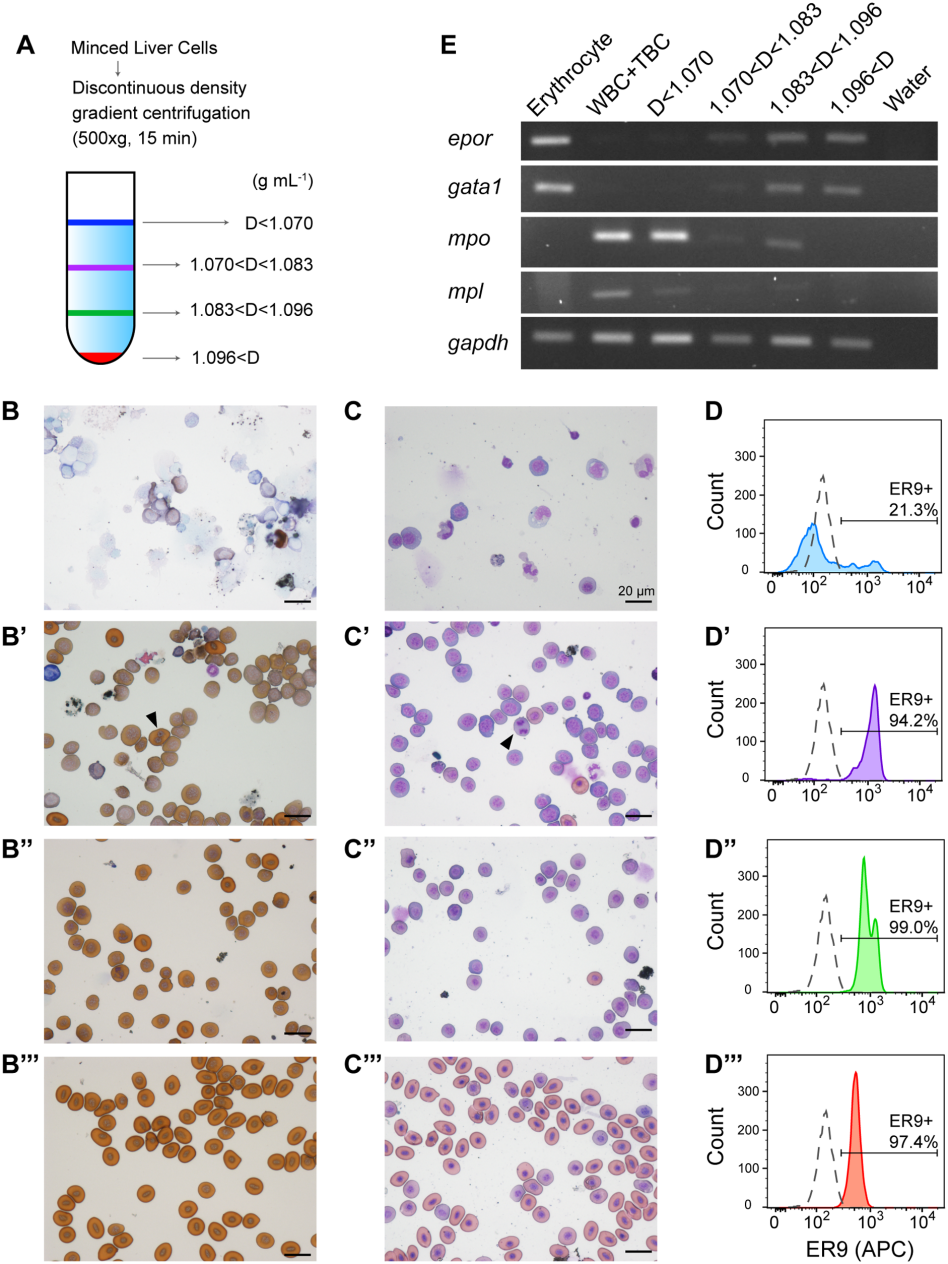
**Reactivity of ER9 to erythroid progenitors.** (A) Illustration of erythroid progenitor fractionation from the liver of *X. tropicalis* using discontinuous density gradient centrifugation. D represents the density (g mL^−1^). (B-C) Images of cells from the liver of *X. tropicalis* fractionated by discontinuous density gradient centrifugation and subjected to chemical staining (×40 magnification). (B) *o*-dianisidine Giemsa staining. (C) May–Grünwald–Giemsa staining. No prime symbol shows D<1.070; single prime shows 1.070<D<1.083; double prime shows 1.083<D<1.096; triple prime shows 1.096<D. (D) Histograms of ER9-APC showing the EPOR expression levels in the indicated fractions of *X. tropicalis* liver cells. Ten thousand cells from each sample were analyzed. (E) RT-PCR analysis for *epor*, *gata1*, *mpo*, *mpl*, and *gapdh* expression in the liver cells fractioned by density gradient centrifugation. Peripheral erythrocytes, white blood cells (WBC), and thrombocytes (TBC) fractioned by density gradient centrifugation were also analyzed as positive control for lineage-specific genes. APC, allophycocyanin; TBC, thrombocyte; WBC, white blood cell.

In mammals, erythroid progenitor cells can be enriched by density gradient centrifugation, removing mature RBCs with high specific density (Nijhof and Wierenga, 1983). Using these approaches, cells obtained from the liver of *X. tropicalis* were separated by Percoll density gradient centrifugation (Fig. 3A). Globin synthesis was observed in populations with specific densities >1.070 g ml^−1^ (Fig. 3B). The intensity of *o*-dianisidine staining increased with increasing density. Cells with higher densities showed the presence of more condensed nuclei than cells with lower density (Fig. 3B-B‴, C-C‴), suggesting an increase in density with differentiation. Populations with specific densities of 1.070–1.083 g ml^−1^ showed the presence of mitotic cells (Fig. 3B′,C′). Each fraction was immunostained with the ER9 antibody and examined by flow cytometry. The fluorescence intensity of ER9-APC could be classified into three levels; the more differentiated the cells, the lower the fluorescence intensity (Fig. 3D). The enrichment of erythroid cells in the populations with specific densities more than 1.070 g ml^−1^ was also confirmed by RT-PCR (Fig. 3E), as assessed by the expression of erythroid-specific genes *epor* and *gata1*. However, granulocyte marker gene *mpo* was also detected in the erythroid fraction, indicating difficulty in complete fractionation depending on the specific density.

### Flow cytometric fractionation of erythroid progenitors using the ER9 antibody and AO staining

**Fig. 4.**
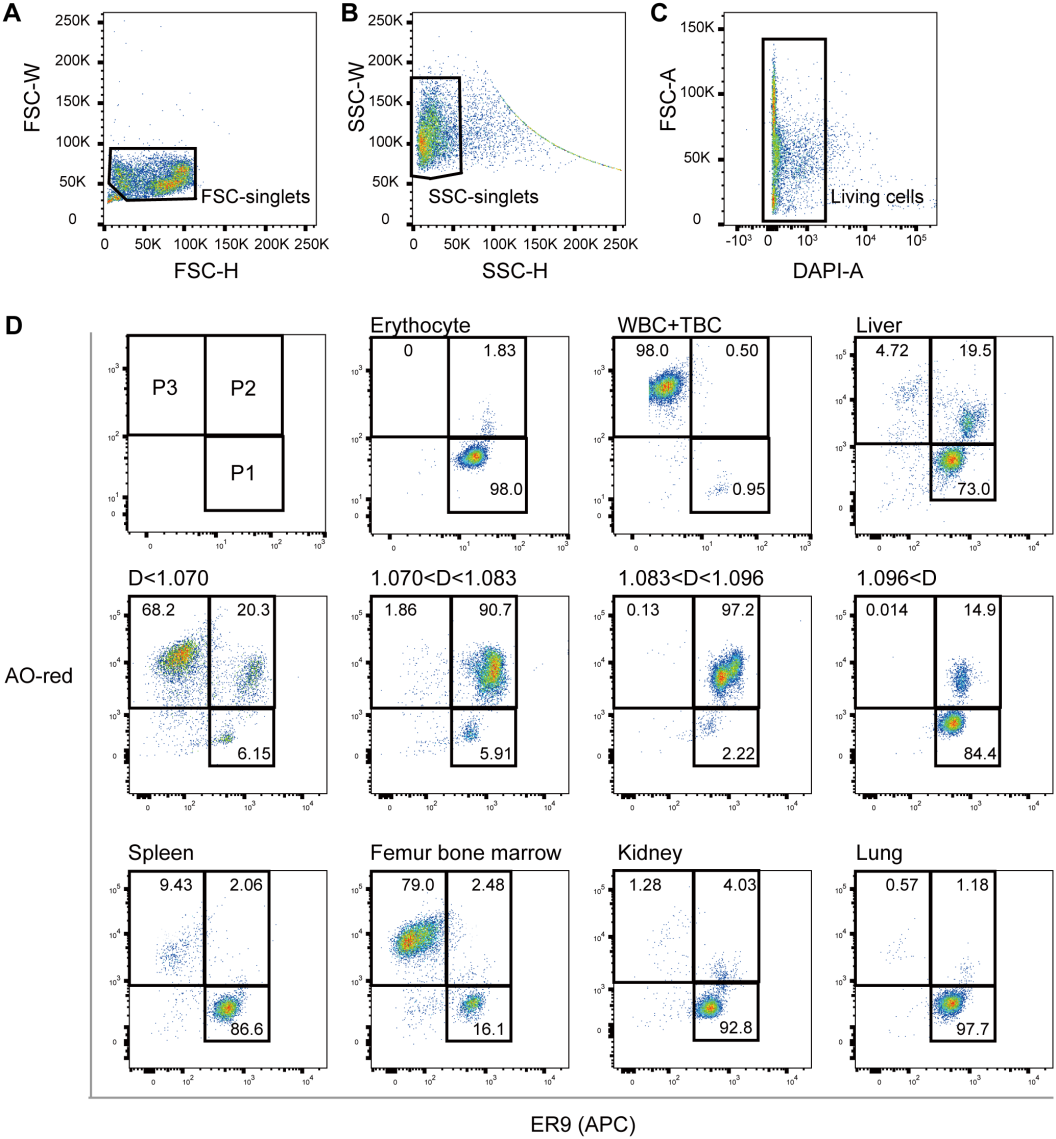
**Gating of fractionation using ER9 and acridine orange.** (A-C) Scatter gram plots and live cell gating before analysis. (A) forward scatter (FSC); (B) side scatter (SSC); (C) 4′, 6-diamidino-2-phenylindole, dihydrochloride (DAPI). (D) ER9 versus acridine orange (AO)-red staining showed the presence of three populations (P1, ER9^+^ AOr^low^; P2, ER9^+^ AOr^high^; P3, ER9^−^ AOr^high^) in the tissues potentially involved in erythropoiesis. Cells from the peripheral blood, liver, spleen, femur bone marrow, kidney, and lung tissues were analyzed. D represents the cell's density (g mL^−1^) fractioned by discontinuous density gradient centrifugation from the liver. The numbers written on the gate indicate the percentage of the population. Ten thousand cells from each sample were analyzed.

ER9 could distinguish erythroid cells from other blood cells; however, fractionating erythroid progenitors and mature erythrocytes in the liver using only ER9 was difficult. Therefore, we added another index. Acridine orange (AO) staining reportedly helps distinguish between mature erythrocytes and other peripheral blood cells in flow cytometry (Sato et al., 2018). AO fluoresces green when bound to double-stranded nucleotides and red when bound to single-stranded nucleotides. AO-red (AOr) staining, single-strand nucleotide, and lysosome volume indicator (Thomé et al., 2016) were used as indexes for erythroid fractionation. After gating out cell debris, doublets, and dead cells (Fig. 4A-C), the liver cells were fractioned into three populations; P1, ER9^+^ AOr^low^; P2, ER9^+^ AOr^high^; P3, ER9^−^ AOr^high^ (Fig. 4D). Peripheral erythrocyte was categorized as ER9^+^ AOr^low^ (Fig. 4D, top). White blood cells and thrombocytes from peripheral blood were classified as ER9^−^ AOr^high^ (Fig. 4B, top). Erythroid progenitors with a specific density greater than 1.070 and less than 1.096 were present in the ER9^+^ AOr^high^ fraction (Fig. 4D, middle).

### Tissue distribution of erythroid progenitors in *X. tropicalis*

Cells from adult organs related to hematopoiesis (spleen, kidney, and bone marrow) were analyzed using ER9 and AO-red as indicators. Furthermore, minced lung tissues were examined because *Xenopus* lung tissues express *epo* at particularly high levels (Nogawa-Kosaka et al., 2010). The ER9^+^ AOr^high^ fraction was dominant in the liver (Fig. 4D). The kidney also had more ER9^+^ AOr^high^ cells than peripheral erythrocytes, suggesting erythropoietic activity (Fig. 4D). We did not analyze adherent cells in these organs; however, the results suggest that the liver is the primary erythropoietic organ in *X. tropicalis* under normal conditions, as in *X. laevis*.

### The morphology of the cells fractioned using ER9 and AO

**Fig. 5.**
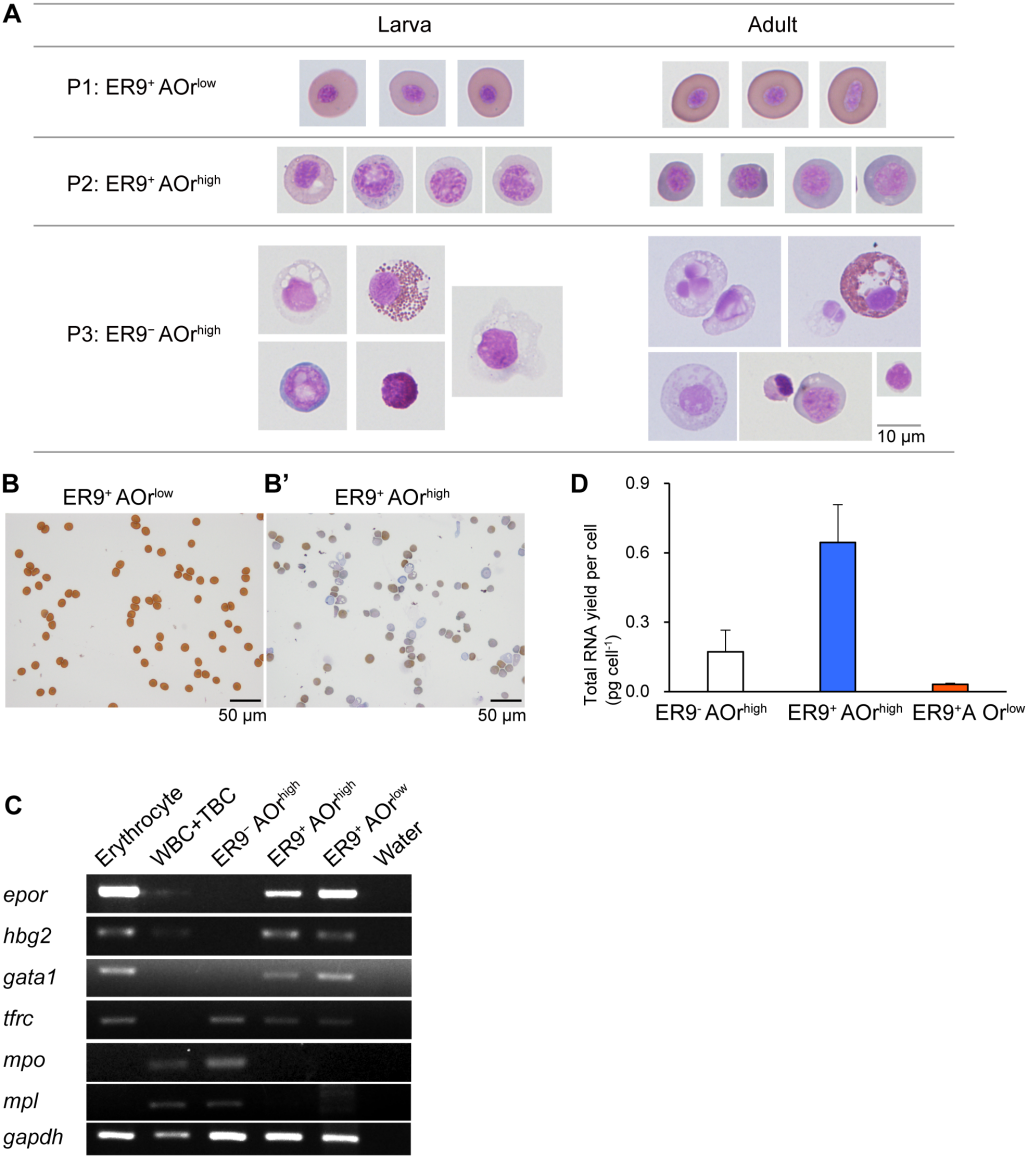
**Images and gene expression of the cells sorted from the liver tissues based on EPOR expression.** (A) Images of the cells in three populations (P1, ER9^+^ AOr^low^; P2, ER9^+^ AOr^high^; P3, ER9^−^ AOr^high^) stained by May–Grünwald–Giemsa method (×60 magnification). (B) ER9^+^ AOr^low^ (no prime symbol) and ER9^+^ AOr^high^ (single prime) cells subjected to *o*-dianisidine Giemsa staining (×20 magnification). (C) RT-PCR results showing RT-PCR analysis for *epor*, *hbg2, gata1*, *tfrc, mpo*, *mpl*, and *gapdh* expression in ER9^−^ AOr^high^, ER9^+^ AOr^low^, and ER9^+^AOr^high^ liver cells. Peripheral erythrocytes, white blood cells (WBC), and thrombocytes (TBC) fractioned by density gradient centrifugation were also analyzed as positive control for lineage-specific genes. (D) Bar graphs showing total RNA (pg) yield per cell in the indicated population. Total RNA was extracted by the acid guanidine-phenol-chloroform method. The bar plot for each parameter with the measured values and SD are shown (*n*=3).

ER9^+^ AOr^low^ cells from the liver were positive for *o-*dianisidine. They showed condensed nuclei, similar to mature erythrocytes (Fig. 5A). The ER9^−^ AOr^high^ cells of the liver showed morphological characteristics of granulocytes and blasts (Fig. 5A). ER9^+^ AOr^high^ cells possess nuclei with open chromatin and less Hb than mature erythrocytes (Fig. 5A-B). These results indicate that erythroid progenitors were enriched in the ER9^+^ AOr^high^ fraction, although it possibly includes cells from other lineages.

### Gene expression of cells fractioned by ER9 antibody and AO

Gene expression was examined in the liver cells to confirm that erythroid lineage cells were enriched in the ER9^+^ fraction. Peripheral erythrocytes, white blood cells, and thrombocytes were analyzed as controls for lineage-specific gene expression. ER9^−^ AOr^high^ cells expressed *mpo, mpl,* and *tfrc,* but not *epor*, *hbg2*, and *gata1*. *tfrc* was not detected in peripheral white blood cells and thrombocytes (Fig. 5C). These results suggest that ER9^−^ AOr^high^ cells are a heterogeneous population that includes granulocyte and thrombocyte lineage cells and other cell types not present in peripheral blood. ER9^+^ cells expressed erythroid marker genes, *epor*, *hbg2*, *gata1,* and *tfrc* (Fig. 5C). There was no significant difference in the expression levels of erythroid marker genes between ER9^+^ AOr^high^ cells and peripheral erythrocytes (Fig. S1), suggesting that the compositional ratios of these gene expressions are maintained before and after erythroid maturation. On the other hand, the yield of total RNA from ER9^+^ AOr^low^ cells (pg per cell) was 21 times lower than that from ER9^+^ AOr^high^ cells (Fig. 5D). This is consistent with the characteristics of mature erythrocytes, where gene expression is suppressed. These results confirmed the enrichment of erythroid lineage cells in the ER9^+^ fraction and the characteristic of mature erythrocytes in ER9^+^ AOr^low^ cells.

### Changes in the number of erythroid cells during metamorphosis

**Fig. 6.**
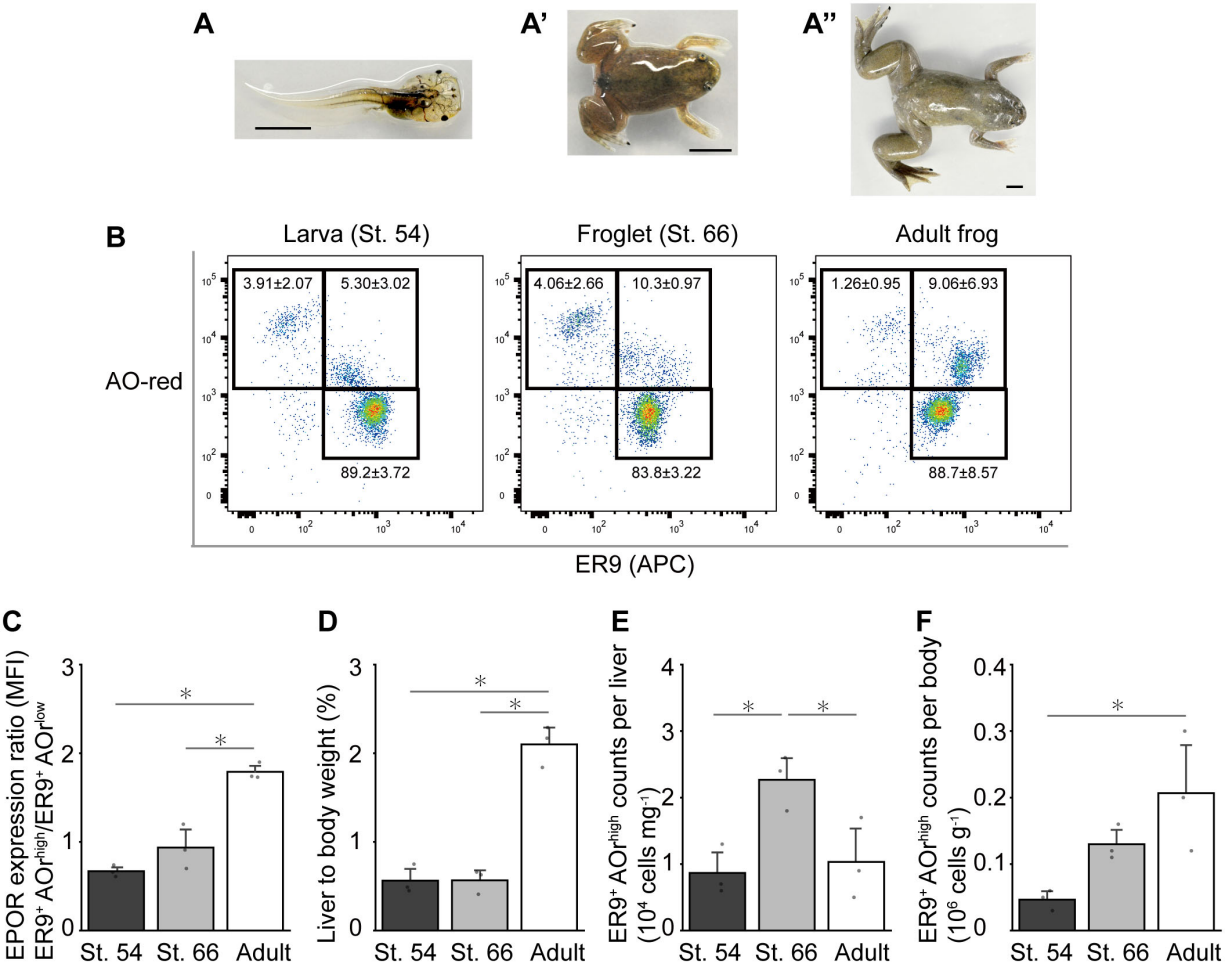
**Comparison of erythroid progenitor counts in the liver at different developmental stages.** (A) Images showing the developmental stages of frogs used in the experiment. No prime symbol shows St. 54 (pre-metamorphosis); single prime shows St. 66 (immediately after metamorphosis); double prime shows adult frog. (B) FACS gating of ER9-APC versus AOr. Left, St. 54; middle, 66; right, adult frogs. The percentages are shown beside each flow-cytogram population with the s.d. (*n*=3). (C) The ratio of the mean fluorescent intensity (MFI) in ER9-APC, (D) liver-to-body weight ratio, (E) ER9^+^ AOr^high^ counts per unit liver weight, and (*F*) ER9^+^ AOr^high^ counts per unit body weight in stage 54, stage 66, and adult frogs. The bar plot for each parameter with the measured values and s.d. are shown (*n*=3). Statistically significant differences are indicated with *P<0.05 (Tukey's test). St, stage; AO, acridine orange; APC, allophycocyanin.

The number of erythroid progenitors was examined in the liver of larvae, froglets, and mature adults. Representative images of each developmental stage are shown in Fig. 6A-A″. There was no significant difference in blood counts (cells L^−1^) between these developmental stages (Fig. S2). Fractionation using ER9 and AO was also applied to cells obtained from froglets and larvae (Fig. 6B). The fluorescence intensity in the adult ER9^+^ AOr^high^ fraction was higher than that in the larval and froglet fractions (Fig. 6C), suggesting differences within the populations. The liver weight-to-BW ratio (LW/BW) of adults was 3.7 times higher than that of froglets (Fig. 6D). ER9^+^ AOr^high^ cell counts per unit of LW were higher in the froglets than in adults and larvae (Fig. 6E). The ER9^+^ AOr^high^ cell counts per unit BW was higher in adults than in larvae and froglets (Fig. 6F). The values are summarized in Table 2.

## DISCUSSION

We established ER9, the monoclonal antibody against *xl*EPOR, in the present study. ER9 recognized the *xl*EPOR (Fig. 1A-B) and neutralized the proliferative effect of *xl*EPO (Fig. 2B). In RT-PCR analysis of *X. tropicalis* liver cells, ER9 positive cells expressed *epor*, and ER9 negative cells did not (Fig. 5C). These results demonstrated ER9 binding to EPOR; however, the specificity of this antibody was not fully demonstrated. This caution is necessary because there were discussions about recognition specificity in the case of antibodies against mammalian EPOR (Elliott et al., 2006). So far, no problematic data about ER9 specificity against EPOR have been available. In future studies, the specificity of ER9 can be discussed in more detail through epitope mapping.

The shift within the histogram of ER9 to erythrocytes (Fig. 1D) is relatively poor compared with that observed in antibodies used for erythroid fractionation in human or mouse model research (Chen et al., 2009; Hu et al., 2013). The poor staining ability of the anti-*xl*EPOR antibody may be attributed to the low EPOR expression, approximately 100–400 molecules, on the surface of primary human and murine cells (Broudy et al., 1991; SASAKI et al., 1987). In mammals, EPOR expression decreases more than 15-fold from proerythroblasts to orthochromatic erythroblasts and is lost in mature red blood cells (Broudy et al., 1991). Previously, we showed that *X. laevis* erythrocytes express *epor* even after maturation (Aizawa et al., 2005). This is the first report on the expression of EPOR on the surface of mature nucleated erythrocytes.

The antigens of ER9 on the erythrocyte membrane were detected as dots (Fig. 1E), consistent with the expression patterns of human EPOR marked with green-fluorescent protein and hemagglutinin of the expression patterns (Ketteler et al., 2002; Tong et al., 2006) and wild-type human EPOR on the membrane of primary erythroid progenitors detected using ^125^I-labeled EPO (Broudy et al., 1991). The ER9 staining patterns may indicate the EPOR expression on the cell membrane.

Our findings suggested a change in EPOR expression between progenitors and mature erythrocytes through metamorphosis (Fig. 6C). This may be attributed to the replacement in the erythroid progenitor population from larval to adult type. Interestingly, froglets show both ER9^high^ and ER9^low^ progenitors in the ER9-AOr fractionation (Fig. 6B). This is consistent with previous reports that show the remaining larval-type erythrocytes in froglets immediately after metamorphosis (Liao et al., 2022; Tamori and Wakahara, 2000b).

During mammalian development, erythropoiesis shifts from the liver to the bone marrow through various distribution mechanisms (Myneni et al., 2021), making it challenging to examine erythropoietic activity in an integrated manner. In the aquatic anuran *X. laevis*, the liver is the dominant erythropoietic organ from the larval to the adult stages (Nogawa-Kosaka et al., 2011; Okui et al., 2016). Therefore, *X. laevis* can be a unique animal model to study changes in RBC production in a single organ during growth. The present study confirmed that the liver is the primary organ for steady-state erythropoiesis in *X. tropicalis* (Fig. 4D), a close diploid relative of *X. laevis*.

Given the 50-fold increase in BW during metamorphosis, the absolute number of erythroid progenitors was compared with the proportion to LW or BW weight (grams). We confirmed loss in LW and BW immediately after metamorphosis, as previously reported (Tata, 1968). Interestingly, the ratio of LW to BW was higher in adults than in larvae and froglets (Fig. 6D). This proportional change may be partly attributed to structural changes in the liver. The organization and size of hepatic cells change during the developmental stages of *X. laevis* (Okui et al., 2016). Similar hepatic reconstruction occurs in the postnatal stage in mammals (Kanamura et al., 1990). However, in the postnatal stage, the ratio of LW to BW of mammals is higher in newborns and lower in adults (Johnson et al., 2005; Vollmar et al., 2002), in contrast to that in *X. tropicalis*. This may reflect the retention of hepatic erythropoiesis in *Xenopus* metamorphosis but a loss in mammalian postnatal alterations.

In parallel with liver growth, the proportional number of ER9^+^ AOr^high^ erythroid progenitors to BW was higher in adults than that in froglets and larvae (Fig. 6F). On the other hand, the proportional number of ER9^+^ AOr^high^ erythroid progenitors to LW was higher in froglets than that in adults and larvae (Fig. 6E). Higher erythropoietic capacity in froglet liver is reasonable because of early demand for adult-type erythrocytes, which is consistent with decreasing LW and removal of larval-type erythrocytes. These results indicate that steady-state erythropoietic activity could change even after the completion of metamorphosis.

In conclusion, the ER9 antibody established in this study is a valuable tool for investigating the function of the EPO-EPOR axis in amphibians. Using this antibody, we demonstrated that the expression of EPOR protein is maintained in *Xenopus* erythroid lineage cells even after maturation. In addition, we showed that metamorphosis alters the erythropoietic activity in the liver. This finding will shed light on how hematopoietic capacity responds to changes in physiological demands due to metamorphosis.

## MATERIALS AND METHODS

### Animals

Adult frogs were bred as described previously (Sato et al., 2018). *Xenopus laevis* Daudin 1982 was purchased from Aquatic Animal Supply (Misato, Japan). Nigerian BH, an inbred line of *Xenopus tropicalis* Gray 1864 (Kashiwagi et al., 2010), was purchased from the National Bioresource Project at Hiroshima University (Hiroshima, Japan). Tadpoles were obtained and raised according to a previously reported method (Okui et al., 2016). The developmental stage of tadpoles was determined based on a previously reported method (Zahn et al., 2022). Only male adult frogs were used in this study. All experimental procedures involving animals were approved by the Regulations for Animal Experimentation at Waseda University, Tokyo, Japan (2010-10A39, 2011-A029, 2012-A029, 2013-A003, 2014-A055, 2015-A001a, 2016-A024, 2017-A001a, 2018-A076, 2019-A074, 2020-A018, 2021-A007, A22-010).

### Generation of monoclonal antibodies against *X. laevis* EPOR

The cDNA fragment encoding the extracellular region of *X. laevis* EPOR.L (*xl*EPOR) was inserted into a mouse IgG2aFc vector pFUSE (#06G07-MT, InvivoGen, San Diego, CA, USA). *xl*EPOR and the Fc region of a mouse IgG fusion protein (*xl*EPOR-Fc) were expressed in human embryonic kidney (HEK) 293T cells using FuGENE HD Transfection Reagent (Roche, Indianapolis, IN, USA). *xl*EPOR-Fc was purified by affinity chromatography with rProtein A sepharose Fast Flow (Cytiva, Tokyo, Japan). Female mice (4 weeks old, BALB/cAjcl line, Japan Clea, Tokyo, Japan) were immunized with *xl*EPOR-Fc (50 μg/100 μl/mouse) with Titer Max Gold (TiterMax, Norcross, GA, USA). Immunization was performed three times every 14 days. Spleen cells of immunized mice and myeloma cell line, PAI cells (JCRB0113, provided from Health Science Research Resource Bank, Japan, Osaka) were fused using the ClonaCell-HY Hybridoma Cloning Kit (STEMCELL Technologies, Vancouver, BC, Canada) according to the manufacturer's instructions. The hybridoma supernatants were screened using enzyme-linked immunosorbent assay for mouse IgG and immunostaining of *xl*EPOR-FDC/P2 cell line. Positive clones were validated by flow-cytometry subjecting *X. laevis* blood cells. Finally, three clones (ER3 derived from JG3, ER9 from CE9, and ER11 from CA11) were selected. The subclass of each antibody was analyzed using an IsoStrip mouse monoclonal antibody isotyping kit (Roche) according to the manufacturer's instructions. The antibodies for erythroid fractionation were purified from the hybridoma culture supernatants using Toyopearl AF-rProtein A HC650F (Tosoh, Tokyo, Japan). The concentration of the antibodies was determined through sandwich ELISA using an anti-mouse IgG (H+L) antibody (#17568, Immuno-Biological Laboratories, Gunma, Japan) and a horseradish peroxidase-labeled secondary antibody (#P0447, Dako, Carpinteria, CA, USA). The chromogenic reaction for ELISA was performed using a peroxidase-coloring kit (Sumitomo Bakelite, Tokyo, Japan) according to the manufacturer's instructions. Sodium dodecyl-sulfate (SDS)-polyacrylamide gel electrophoresis was performed to confirm the presence of a heavy chain and light chain in each MoAb.

### Expression of recombinant *xl*EPO and *X. tropicalis* EPO (xtEPO) in HEK 293T cells

An *xl*EPO.S expression vector was prepared as previously described (Nagasawa et al., 2015; Nogawa-Kosaka et al., 2010). The *xt*EPO-coding sequence was amplified from the lung tissue cDNA using suitable primers (xtepo_cloning_Fw2: ATAGGATCCGCCCCCTACAAGCCTGTC, xtepo_cloning_Re2: TCGGAATTCTCACGTAGATAAGCTTGCCTCGTGG) and the Prime STAR GXL DNA Polymerase (Takara Bio, Shiga, Japan) according to the manufacturer's instructions. PCR products digested by EcoRI and BamHI (Takara Bio) were inserted into pUC19 using the DNA Ligation Kit <Mighty Mix> (Takara Bio). To construct the *xt*EPO expression vector, fragment cDNA amplified from the *xt*EPO-pUC19 vector using suitable primers (xtepo_pfusemIgG2Afc2_fw2: ATACAATGGCAGCCCCCTACAAGCCTGTC, xtepo_pfusemIgG2Afc2_re: TCGGCTAGCTCACGTAGATAAGCTTGCCTCGTGG) and the PrimeSTAR GXL DNA polymerase was digested with NheI and NcoI (Takara Bio) and inserted into the pFUSE-mIgG2Aa-Fc2 vector (InvivoGen, San Diego, CA, USA). The signal peptide was estimated using SignalP version 6.0 (https://services.healthtech.dtu.dk/services/SignalP-6.0/). Each plasmid was sequenced in an ABI3100 Genetic Analyzer (Applied Biosystems, Foster City, CA, USA) using dye-terminator chemistry (BigDye Terminator; Applied Biosystems). The cloned sequence perfectly matched the predicted *X. tropicalis epo*-coding sequence (NCBI Reference Sequence, XM_012954706.3). All constructs were transfected into *Escherichia coli* DH5α (Takara Bio) using CaCl_2_ treatment and heat shock. The plasmids were prepared using the alkaline-SDS lysis method followed by polyethylene glycol-induced precipitation. To obtain recombinant proteins, HEK 293T cells (RIKEN Cell Bank, Tsukuba, Japan) were used as the host. HEK 293T cells were cultured in Dulbecco's Modified Eagle Medium (Nissui Pharmaceutical, Tokyo, Japan) supplemented with 10% fetal bovine serum (Bovogen Biologicals, Victoria, Australia) until 80% confluence was achieved. The culture medium was exchanged with Opti-MEM (Thermo Fisher Scientific, Waltham, MA, USA) before transfection. Aliquots of 9 μg of the plasmids and 54 μg of PEI max (#24765-100, Polysciences, Warrington, PA, USA) were dissolved in 1.5 ml of Opti-MEM, which was then added to the cell culture. The conditioned medium was collected after 4 days of culture and concentrated using centricon YM-3 (Merck Millipore, Burlington, MA, USA). The expression of *xl*EPO and *xt*EPO was confirmed by SDS-PAGE followed by western blotting using anti-*xl*EPO polyclonal antibody (Nagasawa et al., 2015).

### Cell proliferation assay

The biological activity of each recombinant protein and antibody was tested in the cell proliferation assay of IL-3-dependent FDC/P2 cell line or *xl*EPOR-FDC/P2 cell line, which was performed as described previously (Nogawa-Kosaka et al., 2010). The median effective concentration (EC_50_) was determined to normalize the activity of each cytokine, and ten times the EC_50_ was used to test the MoAbs for their ability to inhibit proliferation. Recombinant mouse IL-3 (rmIL-3) was obtained from Kirin Brewery Co. Ltd [lot #YTE1202(100)-34, Tokyo, Japan]. The absorbance in each well was measured using the Synergy H1 microplate reader (Agilent, Santa Clara, CA, USA).

### *Xenopus* cell preparation

Blood cells from adult frogs were prepared as described previously (Sato et al., 2018). Blood cells from larvae and froglets were collected by inserting a glass capillary into the heart. Glass capillary with a length of 90 mm and diameter of 1 mm (GD-1, Narishige Co., Tokyo, Japan) was drawn out with a micropipette puller (PC-10, Narishige Co.). The tip of the capillary was shaved just enough to cause capillary action. Larvae and froglets were anesthetized by 0.2% tricaine methane sulfate (MS222), and their pericardium was removed to expose the ventricles. Then, the capillary coated with EDTA-2Na was inserted into the ventricle, and blood was collected. Liver cells were dispersed with a 27G needle, and cell clumps were removed with a 40 μm nylon mesh. The dispersed cells were washed three times with FACS buffer containing 7/9× Dulbecco's modified phosphate-buffered saline (DPBS), 2% fetal calf serum, and 2 mM EDTA-2Na. In the fractionation of erythroid progenitors, the cells were suspended in 7/9× DPBS with 2 mM EDTA-2Na (PBS-E) and carefully laid on Percoll (Cytiva, Tokyo, Japan) discontinuous density gradients. The Percoll solutions were adjusted to 7/9× DPBS with 2 mM EDTA-2Na. Cells were centrifuged at 500×***g*** for 15 min at 22°C. White blood cells, thrombocytes, and other cell types were obtained from peripheral blood at a density <1.083 g ml^−1^, the erythrocytes were obtained at a density >1.083 g ml^−1^.

### Flow cytometric analysis

The FDC/P2 or *xl*EPOR-FDC/P2 cell line analysis used monoclonal antibodies biotinylated by Biotin-(AC_5_)_2_-OSu (Dojindo, Kumamoto, Japan). Antibody solutions were diluted with FACS buffer containing 1×DPBS, 2% fetal calf serum, and 2 mM EDTA-2Na. Before the antibody reaction, cells were incubated with FACS buffer containing 0.8% mouse serum (Dako) to avoid non-specific antibody binding by the Fc receptor. Cells were incubated on ice with each biotinylated antibody at 2 μg ml^−1^ for 40 min. After washing, cells were incubated on ice with Streptavidin Alexa fluor 680 solutions diluted ×200 (Thermo Fisher Scientific) for 30 min. Flow cytometric analysis was performed on the BD FACS Aria IIIu (BD Biosciences, San Jose, CA, USA).

In the analysis of *Xenopus* cells, antibody solutions were diluted with FACS buffer containing 7/9×DPBS, 2% fetal calf serum, and 2 mM EDTA-2Na. Cells were incubated on ice with each antibody at 5 μg ml^−1^ or the recommended concentration. The mouse IgG2a isotype control (Tonbo Bioscience, San Diego, CA, USA) was used as the negative control. Cells were stained with 5 μg ml^−1^ anti-mouse IgG goat antibody conjugated with allophycocyanin (APC) (#ab130782, Abcam, Cambridge, UK). Cells were incubated with 5 μg ml^−1^ acridine orange (AO; Sigma Aldrich, St. Louis, MO, USA) for 15 min and 1 μg ml^−1^ DAPI (Dojindo, Kumamoto, Japan) for 5 min at room temperature. Before analysis, cells were washed thrice with FACS buffer, and clumps were removed using a 40 μm nylon mesh. The reactivity of each MoAb to *Xenopus* EPOR was evaluated using an FC500 flow cytometer (Beckman Coulter, Brea, CA, USA). Erythroid progenitors were fractioned using the BD FACS Aria IIIu.

All flow cytometric results were analyzed using the FlowJo software v10.4.2 (FlowJo LLC, Ashland, OR, USA).

### Cytological analysis

Centrifuged smears were prepared using Cytopro 7620 (Wescor, Logan, UT, USA). As previously reported, the smears were stained with the May–Grünwald–Giemsa solution or *o*-dianisidine–Giemsa (Nogawa-Kosaka et al., 2010; Okui et al., 2013). Cytological images were obtained using light microscopy (model BX51; Olympus, Tokyo, Japan).

### Reverse transcription polymerase chain reaction (RT-PCR)

Total RNA was extracted from various tissues of *X. tropicalis* using the acid guanidine-phenol-chloroform (AGPC) method (Chomczynski and Sacchi, 1987) with some modifications. In this study, the AGPC solution was composed of 40.7% (w/v) phenol, 2.8 mM 8-quinolinol, 0.1 M sodium acetate, 5% (v/v) glycerol, 415 mM ammonium thiocyanate, and 0.8 M guanidine thiocyanate. The purified total RNA was reverse-transcribed into cDNA using ReverTraAce (Toyobo, Osaka, Japan) with oligo-(dT)_16_ according to the manufacturer's instructions. PCR was performed using OneTaq (New England Biolabs, Ipswich, MA, USA) according to the manufacturer's instructions, and images of agarose gels were acquired with the LAS-3000 image analysis system (Fujifilm, Tokyo, Japan). The sequences of the primers used for RT-PCR are provided in Table 1.

**Table 1.**
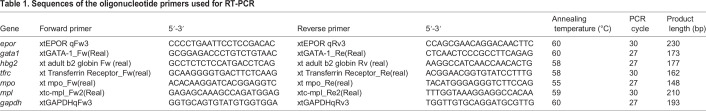
Sequences of the oligonucleotide primers used for RT-PCR

**Table 2.**
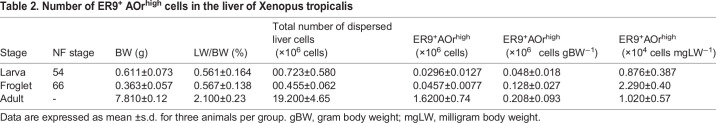
Number of ER9^+^ AOr^high^ cells in the liver of Xenopus tropicalis

Real-time PCR analyses were performed on a StepOnePlus instrument (Thermo Fisher Scientific) using Taq Pro Universal qPCR Master Mix (Vazyme, Nanjing, China) and analyzed by StepOnePlus software (Thermo Fisher Scientific).

### Statistical analysis

The experimental data shown in the bar graph or dot plots represent the mean ±standard deviation (s.d.). The statistical significance of the data was evaluated using one-way analysis of variance. *P* values less than 0.05 were considered statistically significant. Statistical analysis was performed using IBM SPSS Statistics 28 (IBM, Armonk, NY, USA).

## Supplementary Material

10.1242/biolopen.059862_sup1 Supplementary information
