# Supplementary material for: Isolation and evaluation of erythroid progenitors in the livers of larval, froglet, and adult Xenopus tropicalis
Source: Biol Open. 2023 Jul 27;12(8):bio059862. doi: 10.1242/bio.059862 (PMC10399205; doi:10.1242/bio.059862)
Supplement: Supplementary information [file biolopen-12-059862-s1.pdf]

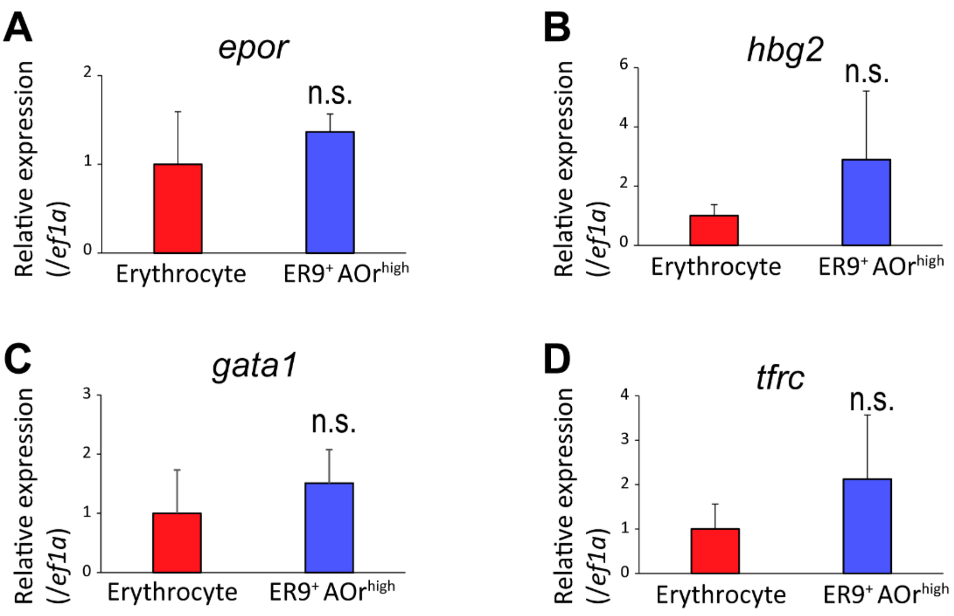


**Fig. S1 (related to Fig. 5). Real-time PCR analysis comparing peripheral erythrocytes and $ER9^{+}AOr^{high}$ liver cells.** A, *epor*; B, *hbg2*; C, *gata1*; D, *tfrc*. n.s. shows there is no significant difference (n=3, $p < 0.05$, Tukey's test).

Biology Open • Supplementary information

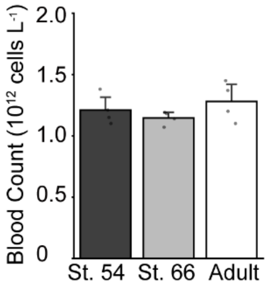


**Fig. S2 (related to Fig. 6). Comparison of blood count (cells $mL^{-1}$) between larvae (St. 54), froglets (St. 60), and adults.** The bar plot for each parameter with the measured values and SD are shown ($n = 4$). There is no significant difference between each developmental stage ($n=4$, $p < 0.05$, Tukey's test).
